# Beam control system and output fine-tuning for safe and precise delivery of FLASH radiotherapy at a clinical linear accelerator

**DOI:** 10.3389/fonc.2024.1342488

**Published:** 2024-01-18

**Authors:** Elise Konradsson, Pontus Wahlqvist, Andreas Thoft, Börje Blad, Sven Bäck, Crister Ceberg, Kristoffer Petersson

**Affiliations:** ^1^ Medical Radiation Physics, Department of Clinical Sciences, Lund University, Lund, Sweden; ^2^ Radiation Physics, Department of Hematology, Oncology and Radiation Physics, Skåne University Hospital, Lund, Sweden; ^3^ Oxford Institute for Radiation Oncology, Department of Oncology, University of Oxford, Oxford, United Kingdom

**Keywords:** FLASH-RT, ultra-high dose rate, beam control, clinical linear accelerator, electrons

## Abstract

**Introduction:**

We have previously adapted a clinical linear accelerator (Elekta Precise, Elekta AB) for ultra-high dose rate (UHDR) electron delivery. To enhance reliability in future clinical FLASH radiotherapy trials, the aim of this study was to introduce and evaluate an upgraded beam control system and beam tuning process for safe and precise UHDR delivery.

**Materials and Methods:**

The beam control system is designed to interrupt the beam based on 1) a preset number of monitor units (MUs) measured by a monitor detector, 2) a preset number of pulses measured by a pulse-counting diode, or 3) a preset delivery time. For UHDR delivery, an optocoupler facilitates external control of the accelerator’s thyratron trigger pulses. A beam tuning process was established to maximize the output. We assessed the stability of the delivery, and the independent interruption capabilities of the three systems (monitor detector, pulse counter, and timer). Additionally, we explored a novel approach to enhance dosimetric precision in the delivery by synchronizing the trigger pulse with the charging cycle of the pulse forming network (PFN).

**Results:**

Improved beam tuning of gun current and magnetron frequency resulted in average dose rates at the dose maximum at isocenter distance of >160 Gy/s or >200 Gy/s, with or without an external monitor chamber in the beam path, respectively. The delivery showed a good repeatability (standard deviation (SD) in total film dose of 2.2%) and reproducibility (SD in film dose of 2.6%). The estimated variation in DPP resulted in an SD of 1.7%. The output in the initial pulse depended on the PFN delay time. Over the course of 50 measurements employing PFN synchronization, the absolute percentage error between the delivered number of MUs calculated by the monitor detector and the preset MUs was 0.8 ± 0.6% (mean ± SD).

**Conclusion:**

We present an upgraded beam control system and beam tuning process for safe and stable UHDR electron delivery of hundreds of Gy/s at isocenter distance at a clinical linac. The system can interrupt the beam based on monitor units and utilize PFN synchronization for improved dosimetric precision in the dose delivery, representing an important advancement toward reliable clinical FLASH trials.

## 1 Introduction

Given the promising preclinical findings of using ultra-high dose rates (UHDR) radiation delivery, FLASH radiotherapy (FLASH-RT) is approaching a clinical translation (1–4). Recently, several commercial medical linear accelerators (linacs) dedicated for UHDR delivery of electron beams have been developed, such as the Mobetron by IntraOp and the FLASHknife/Oriatron eRT6 by THERYQ (5, 6). However, these irradiation devices are only available to a few institutions. To make it more feasible for a broader scientific community to investigate the potential advantages and challenges of FLASH-RT in a clinical context, several institutions have adapted their clinical linacs to accommodate UHDR electron beams (7–11). However, as these linacs are not originally designed for UHDR delivery, they face several technical challenges related to beam control, safety, stability, and precision in the delivery.

FLASH-RT involves extremely short treatment times, generally less than 0.2 s (12), necessitating a robust dosimetry system with high temporal resolution for real-time dose monitoring and beam control. Conventionally, transmission monitor chambers mounted in the beam path are used for monitoring and controlling radiation delivery in radiotherapy, including beam interruption upon reaching the desired dose. Nevertheless, when exposed to UHDR, ionization chambers experience recombination effects that have limited their usefulness for FLASH-RT (13, 14). Recent research has demonstrated that these recombination effects can be mitigated by increasing the chamber voltage and reducing the electrode spacing (15, 16). Furthermore, the remaining effects can be corrected for by modeling the ion collection efficiency as a function of the mean dose-per-pulse (DPP) (17, 18). This approach holds the potential to render transmission monitor chambers useful for monitoring UHDR beams. However, to ensure safety during UHDR delivery, supplementary independent systems should be employed for beam interruption.

Several adapted linacs have encountered difficulties in achieving a stable and precise delivery (7, 8, 19) If the accelerator is mistuned, the initial pulse (or pulses) may deliver a lower dose compared to the subsequent pulses. For instance, Schuler et al. reported low DPP values in the first few pulses (7), and Rahman et al. observed a ramp-up period for the initial 4-5 pulses delivered by a Varian 21EX, resulting in significant underdosage (19). In UHDR delivery, these characteristics can substantially influence the temporal structure of the delivery and potentially affect the biological effect. Another feature of clinical linacs adapted for UHDR delivery is that the prescribed doses are restricted to discrete values, and that adjusting the DPP within the UHDR regimen in an easy and practical way can be challenging.

In 2019, we described the procedure for modifying a clinical Elekta Precise linac (Elekta AB, Stockholm, Sweden) to enable the delivery of a 10 MeV electron beam with average dose rates exceeding 120 Gy/s at the cross-hair foil, over 250 Gy/s at the multi-leaf collimator, and beyond 1000 Gy/s at the wedge position (8). Subsequently, we have undertaken a series of continuous upgrades, carried out preclinical investigations (20), and conducted clinical trials involving canine patients (21, 22) using this modified linac.

As a step towards preparing the accelerator for future human clinical trials, we introduce an upgraded beam control system capable of delivering UHDR electrons at the treatment isocenter, based on monitor units (MUs) rather than the number of pulses. We also present a method to ensure the stability and reproducibility of the UHDR beam. Moreover, this system allows for output adjustment within the first pulse by synchronizing the delivery with the accelerator’s pulse forming network (PFN), permitting a more dosimetrically precise dose delivery.

## 2 Materials and methods

### 2.1 Modifications to the clinical linear accelerator

**Figure 1:**
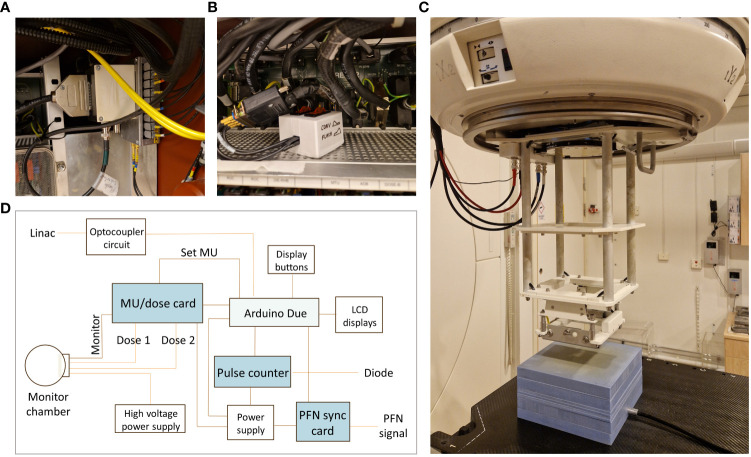
Modifications to the clinical linear accelerator. **(A)** For UHDR irradiation, an optocoupler is connecting an external beam control system to the linac, making is possible to enable or disable the thyratron trigger signals. **(B)** The signal from the linac’s built-in monitor chamber is disabled using a switch. **(C)** In the current setup, an external monitor chamber was reconfigured and mounted in the upper part of an electron applicator. Measurements were performed with EBT3 film positioned at 2.2 cm depth and an ion chamber positioned at 9 cm depth in a 10 cm solid water phantom. **(D)** A schematic illustration of the components in the upgraded external in-house built beam control system.

We have previously described the process of adapting a clinical linac (Elekta Precise, Elekta AB, Stockholm, Sweden) to enable an UHDR 10 MeV electron beam (8). In clinical mode, the accelerator generates 3.5 µs electron pulses and is operated at a pulse repetition frequency (PRF) of 200 Hz. With our modifications, the linac can be temporarily shifted between clinical mode and UHDR mode, where its pulse delivery is controlled through an in-house built external beam control system. This is achieved by using an optocoupler, making it possible to enable or disable the thyratron trigger signals to the linac (**Figure 1A**). In addition, the signal from the built-in monitor chamber is disabled using a switch (**Figure 1B**) to avoid the accelerator being interrupted by the high dose rate. Furthermore, the linac is operated without the primary and secondary scattering foils in the beamline. The initial modifications and beam control system were described by Lempart et al. (8). Following that report, we have made successive improvements. In the setup described in this paper, an external transmission ionization chamber (Elekta AB, Stockholm, Sweden) was reconfigured and mounted in the upper part of a 10x10 cm^2^ electron applicator, 55 cm from the target reference point (**Figure 1C**). The chamber has two dose channels, each with an electrode distance of 0.6 mm, and was operated with an applied chamber voltage of 850 V.

### 2.2 Measurement setup

To determine the absorbed dose and lateral dose profiles, we used EBT3 film (Ashland Specialty Ingredients G.P., Bridgewater, New Jersey, USA), and for relative beam output measurements during tuning, we used a 0.6 cm^3^ Baldwin-Farmer type ion chamber (NE-2505/3-3A).

The measurement setup consisted of a 10 cm Solid Water HE phantom (Gammex Inc., Middleton, Wisconsin, USA) at a source-to-surface distance (SSD) of 100 cm. EBT3 film was positioned at the depth of dose maximum (2.2 cm depth), and the ion chamber was positioned at 9 cm depth in the bremsstrahlung tail of the electron beam, where the dose is 1.5% of the peak dose and the recombination effect is negligible (**Figure 1C**). The EBT3 film batch was calibrated under reference dosimetry conditions at conventional dose rates in a clinical 10 MeV beam using the same linear accelerator, against a plane parallel ionization chamber, traceable to a secondary standard laboratory (National Metrology Laboratory, Solna, Sweden). For calibration, 20 dose levels from 0-30 Gy were established using two film measurements for each level (**Supplementary Figure 1**).

### 2.3 Beam tuning

When using the accelerator in UHDR-mode, the servo control items for the electron gun filament current (gun standby, gun aim and gun current) were manually adjusted to 7.5 A to maximize the output. This value was based on our previously published data by Lempart et al. and confirmed by repeated measurements of the relative output as a function of the gun filament current. Following our previous report, the beam tuning process has been further improved. To further tune the beam for maximal output, the influence of the physical tuner that compensates for changes in the magnetron frequency (tuner rest value) was investigated. The optimal setting was determined by measuring the relative output at different tuner rest values. The relative output was measured with the Farmer-type ion chamber at 9 cm depth in the solid water phantom. Subsequently, a range of +/- 5 from the nominal tuner rest value was chosen to fine tune the beam at the beginning of each day of use in UHDR mode.

As a measure of the stability of the UHDR delivery, repeated output measurements were performed with radiochromic film after beam tuning. The repeatability was determined by one film measurement following a 10-pulse delivery (mean DPP ~0.85 Gy) each minute for 20 consecutive minutes. In addition, the reproducibility was estimated by film measurements following fifteen 10-pulse deliveries distributed over five different days over a period of 3 months. To estimate the variation in DPP, a film strip was attached to a circular nozzle on an electric motor operated at 3800 rpm and simultaneously irradiated with 10 pulses using a 1x1 cm^2^ field.

### 2.4 Beam control system

To improve the safety and accuracy of the UHDR delivery at the clinical linear accelerator, a novel system for beam control was designed and evaluated. A schematic illustration of the components in the beam control box is presented in **Figure 1D**. The system was designed to interrupt the beam based on: 1) a preset number of MU, 2) a preset number of pulses, or 3) a preset delivery time. Until any of these conditions are met, an Arduino Due microcontroller (Atmel Corporation, San Jose, CA, USA) sends a signal to the optocoupler, allowing the trigger pulses to reach the thyratron of the linac. The microcontroller has a clock speed of 84 MHz and a maximum ACD sampling frequency of 1 MHz. Following each delivery, the delivered number of MU, the number of pulses delivered, the time from the start of the first pulse to the end of the last pulse, as well as the estimated PRF are displayed on the beam control system, allowing the user to receive feedback on the temporal structure of the delivery. The system also displays whether the beam was interrupted based on MU, pulses, or time. The beam control system consists of three modules; a MU/dose module, a pulse counter module, and a PFN synchronization module, described in more detail below.

#### 2.4.1 Monitor unit/dose module

The system was designed to primarily interrupt the beam based on the charge measured by a monitor detector, i.e., in this study the external transmission ionization chamber. The MU module integrates the collected signal (from both channels), calculates a corresponding MU value, and compares it to the preset MU following each pulse. The beam is interrupted when the calculated MU exceeds the preset MU. In this paper we used one of the dose channels of the reconfigured monitor chamber mounted in the upper part of a 10x10 cm^2^ electron applicator. Following each electron pulse, the signal is collected and corrected for temperature and pressure. Due to dose-rate dependent recombination effects in the chamber, a recombination correction strategy [as shown in our previous work (17, 18)] was applied. This involved determining the ion collection efficiency (ICE), i.e., the inverse of the recombination correction factor, after each pulse using a logistic model of the ICE as a function of the apparent DPP (Q/C), established for the specific chamber and dose channel:


$$ICE=\frac{1}{{\left[1+{\left(\frac{Q}{C}*10^{4}*\frac{d^{2}}{V}\right)}^{\gamma }\right]}^{\delta }}$$

where Q is the chamber signal per pulse in nC (corrected for temperature and pressure), C is a calibration coefficient in nC/Gy determined with EBT3 film measurements at conventional dose rates, d is the electrode distance in mm, V is the chamber voltage in volt, and γ and δ are fitting parameters with no clear physical interpretation. The corrected signal was normalized such that 100 MU corresponds to 1 Gy in the reference geometry (i.e., 2.2 cm depth at SSD=100 cm). The linearity between the calculated number of MU and the dose measured with film in the solid water phantom in the reference geometry was investigated by five measurements for each of the pre-set number of pulses: 3, 6, 9, 12, 15, 18, 21, and 24. The absorbed dose in the reference geometry was determined with EBT3 film. The monitor chambers second dose channel can be used in a similar way to allow the interruption based on either of the channels.

#### 2.4.2 Pulse counter module

As a secondary system, the irradiation can be interrupted based on a preset number of electron pulses. The pulse counter module counts the delivered number of pulses using a diode (PIN-type, EDD 2-3G Diode, IBA Dosimetry GmbH, Schwarzenbruck, Germany) positioned at the edge of the radiation field (not to impact the field homogeneity). The pulses are digitalized and counted by the microcontroller unit by measuring the rising flanks of the signal (see Lempart et al. (8) for details). The number of pulses counted by the diode was verified against the number of pulses detected by the external monitor chamber connected to a digital oscilloscope (model RBT2004, Rohde & Schwarz, Munich, Germany). As an additional safety feature, the delivery will also be interrupted based on time. If the preset number of pulses is n, the equipment will only allow the accelerator to irradiate for (n-1)/PRF seconds before the triggering pulses to the thyratron are interrupted, ensuring that no more than the set number of pulses will be delivered.

#### 2.4.3 Pulse forming network synchronization

When initiating “beam on” on the Elekta Precise linac, there is a ramp-up of the PRF such that the first few pulses are delivered with a lower rate. For UHDR radiotherapy, where the number of pulses is greatly reduced compared to radiotherapy at conventional dose rates, this will have a large impact on the treatment time. To address this issue, the PFN sync module was constructed and connected to the accelerator’s PFN to monitor its charging cycle. To avoid the ramp-up period, a delay time can be added from the first detection of the PFN signal until the trigger pulses are allowed to pass the optocoupler. The delay time is adjustable to release the first trigger pulse at any point during the PFN upload, thereby adjusting the charge in the first pulse. To investigate how the output in the first pulse depends on the PFN delay time, the relative output for a 1-pulse delivery was measured with the Farmer-type chamber at 9 cm depth in the solid water phantom, for different delay times between 200 and 720 ms in steps of 20 ms.

#### 2.4.4 Evaluation of beam control system

The method of using the PFN charge information to increase the precision in the dose delivery was investigated by delivering the first pulse when the PFN charge was not fully loaded. In this paper, precision is defined as the ability to deliver a preset number of MU. The desired number of MU was set between 250 and 2500 MU in steps of 250 MU, and five measurements were performed at each level with the PFN delay time set to target the corresponding MU value. The preset number of MU was compared to the delivered number of MU.

To assess the beam control system under different simulated failure modes, the ability of the three systems for beam interruption (monitor detector, pulse counter, and timer) were studied independently. For this part, the PFN delay time was adjusted to target a preset number of 500 MU, the number of pulses (n) was set to 7 (determined as the prescribed dose divided by the expected mean DPP, with one extra pulse added due to the adjusted PFN delay time resulting in a reduced mean DPP). First, the ability of the monitor detector to interrupt the beam in a simulated case of pulse counter failure was tested by moving the pulse counting diode from the radiation field. Secondly, the ability of the pulse counter to interrupt the beam in a simulated case of monitor detector failure was tested by disabling the chamber voltage. Thirdly, the ability of the timer to interrupt the beam after 30 ms, i.e. (n-1)/200, simulating both a pulse counter and monitor detector failure was tested by both disabling the chamber voltage and removing the pulse counting diode from the radiation field. For each test, 10 beam deliveries were performed.

## 3 Results

### 3.1 Beam tuning

**Figure 2:**
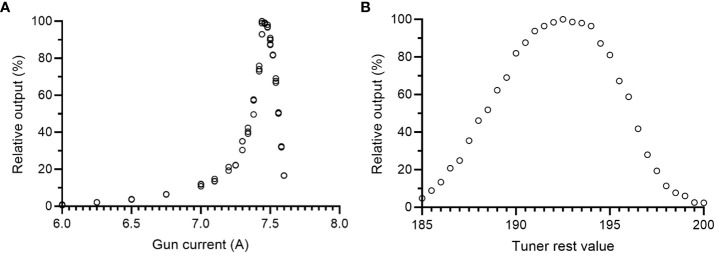
Beam fine-tuning of the 10 MeV electron beam. Relative output measurements for different **(A)** gun current values and **(B)** tuner rest values. Relative output measurements were performed with a Farmer-type ion chamber positioned in the bremsstrahlung tail of the electron beam (9 cm depth). Maximum relative output was achieved with a gun current around 7.5 A and a tuner rest value of 192.5.

**Figure 3:**
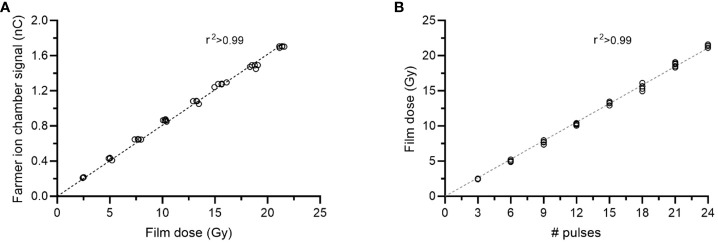
Characteristics of the dose delivery at UHDR. There was a linear relation (r2>0.99) between the absorbed dose measured with film at 2.2 cm depth and **(A)** the signal from the Farmer-type ion chamber positioned at 9 cm depth, as well as **(B)** the number of pulses delivered.

**Figure 4:**
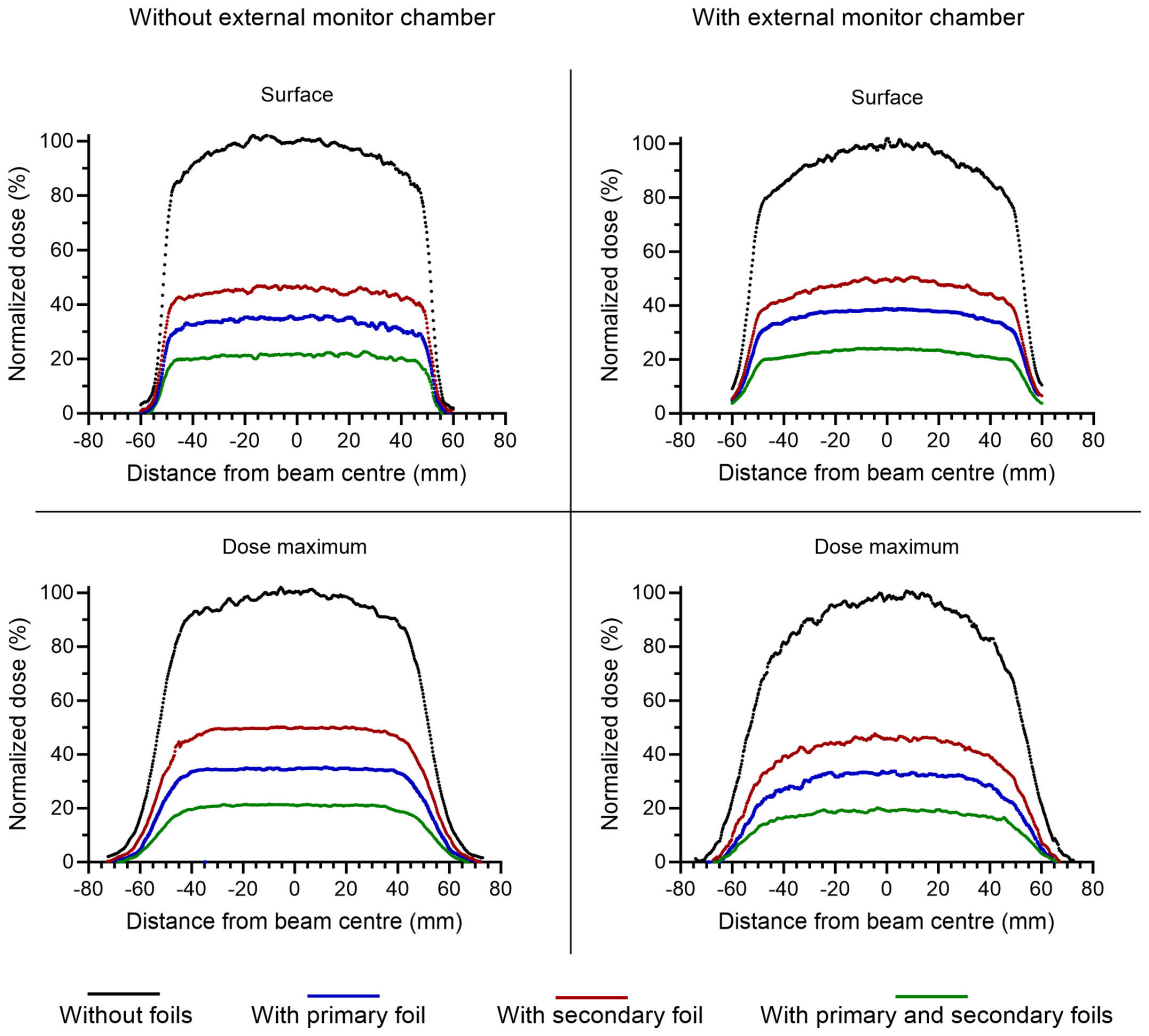
Crossline dose profiles measured with radiochromic film at the surface (upper panels) and at the depth of dose maximum (lower panels), with (right panels) or without (left panels) an external monitor detector in the beam path. The different colors represent different configurations of scattering foils, with black representing no filters, blue representing the primary foil, red representing the secondary foil, and green representing both foils. The dose is normalized with respect to the configuration without scattering foils.

The settings for the gun filament current and the physical tuner had a large influence on the output. Measurements of the relative output at different gun current settings confirmed that 7.44-7.50 A resulted in the highest relative output, as measured using the Farmer-type ion chamber positioned in the bremsstrahlung tail of the electron beam (**Figure 2A**). In this position, the Farmer-type chamber was linear (r^2^>0.99) with the absorbed dose measured with EBT3 film at 2.2 cm depth (**Figure 3A**). At this gun current setting, the accelerator could be operated with tuner rest values ranging between 185 and 200. The highest achievable relative output was found at a tuner rest value of 192.5 (**Figure 2B**), with a corresponding absorbed dose of 0.87 Gy/pulse or 1.05 Gy/pulse at dose maximum at isocenter distance (SSD = 100 cm), with or without the external monitor chamber in the field. As a comparison, by reintroducing the primary and secondary scattering foil into the beams path, the output at dose maximum was reduced by ~80% compared to the output without foils. By only introducing one of the foils, the reduction was ~65% or ~50% for the primary and secondary foil, respectively. With the 10x10 cm^2^ electron applicator, the beam flatness (defined as the maximum percentage variation from the average dose over a given area) at the surface was within 5% over an area of 5.5 cm^2^ or 7.2 cm^2^, with or without the monitor chamber in the field (**Figure 4**). With both filters reintroduced, this area could be increased to 6.5 cm^2^ or 7.8 cm^2^, respectively.

**Figure 5:**
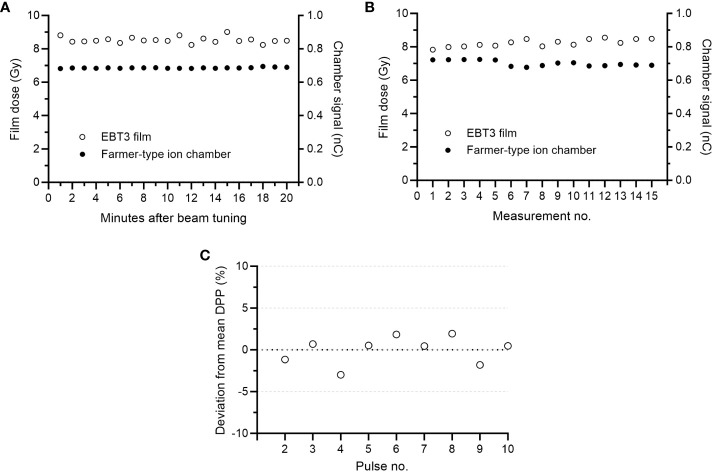
**(A)** The variation in absorbed dose measured with film and Farmer-type ion chamber following one 10-pulse delivery each minute for 20 minutes (standard deviation (SD) of 2.2% and 0.4%, respectively). **(B)** The variation in absorbed dose for fifteen 10-pulse measurements performed over a 3-month period (SD of 2.6% for film measurements and 2.4% for Farmer-type ion chamber measurements). **(C)** The variation in dose-per-pulse (DPP) was estimated by attaching a film strip to an electric motor. Excluding the first pulse, the estimated SD in DPP was 1.7%.

With a tuned beam, the absorbed dose measured using EBT3 films positioned at 2.2 cm depth at an SSD of 100 cm was linear (r^2^>0.99) with the number of pulses delivered (**Figure 3B**). The SD of five film measurements was <2.5% for delivering 3, 6, 9, 12, 15, 18, 21 or 24 number of pulses. The absorbed dose divided by the number of pulses for the total of 40 measurements was 0.86 ± 3.2% (mean ± SD). The repeatability measurements showed a stable output following beam tuning. The film dose measured for one 10-pulse delivery, each minute for 20 minutes, was 8.5 ± 2.2% (**Figure 5A**). The corresponding SD in the Farmer-type ion chamber reading was 0.4%. The output was also stable over a period of 3 months. The 15 measurements performed during this period resulted in a SD of 2.6% for the absorbed dose measured by film, and 2.4% for the signal measured by the Farmer-type ion chamber (**Figure 5B**). The variation in DPP was estimated from a film strip attached to an electric motor (**Supplementary Figure 2**). The first pulse was excluded from the analysis. The deviations from the mean DPP were <3% (**Figure 5C**), with a SD of 1.7%.

### 3.2 Beam control system

**Figure 6:**
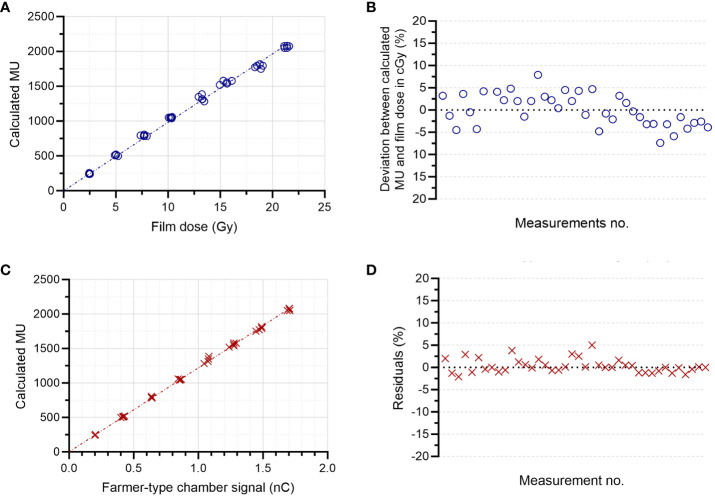
Determination of the delivered number of monitor units (MUs). There was a linear relation (r2>0.99) between the MUs determined by the external monitor chamber and **(A)** the absorbed dose in cGy determined by film at 2.2 cm depth, with a mean absolute deviation of 3% **(B)**. There was also a linear relation (r2>0.99) between the MUs determined by the external monitor chamber and **(C)** the signal from the Farmer-type ion chamber at 9 cm depth, with a mean absolute error of 1.1% **(D)**.

**Figure 7:**
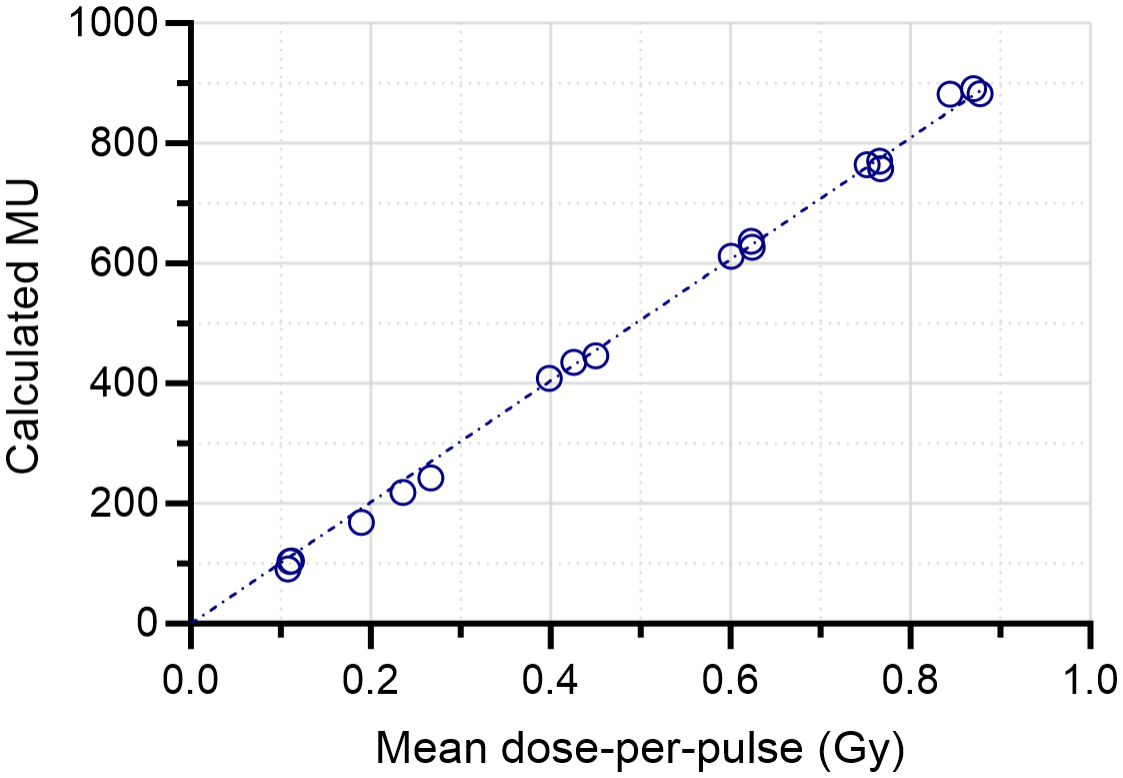
Linear relation (r^2^>0.99) between the delivered number of monitor units (MUs) and the mean dose-per-pulse (DPP) measured with film following a 10-pulse delivery. The mean DPP was measured at 2.2 cm depth in a solid water phantom.

**Figure 8:**
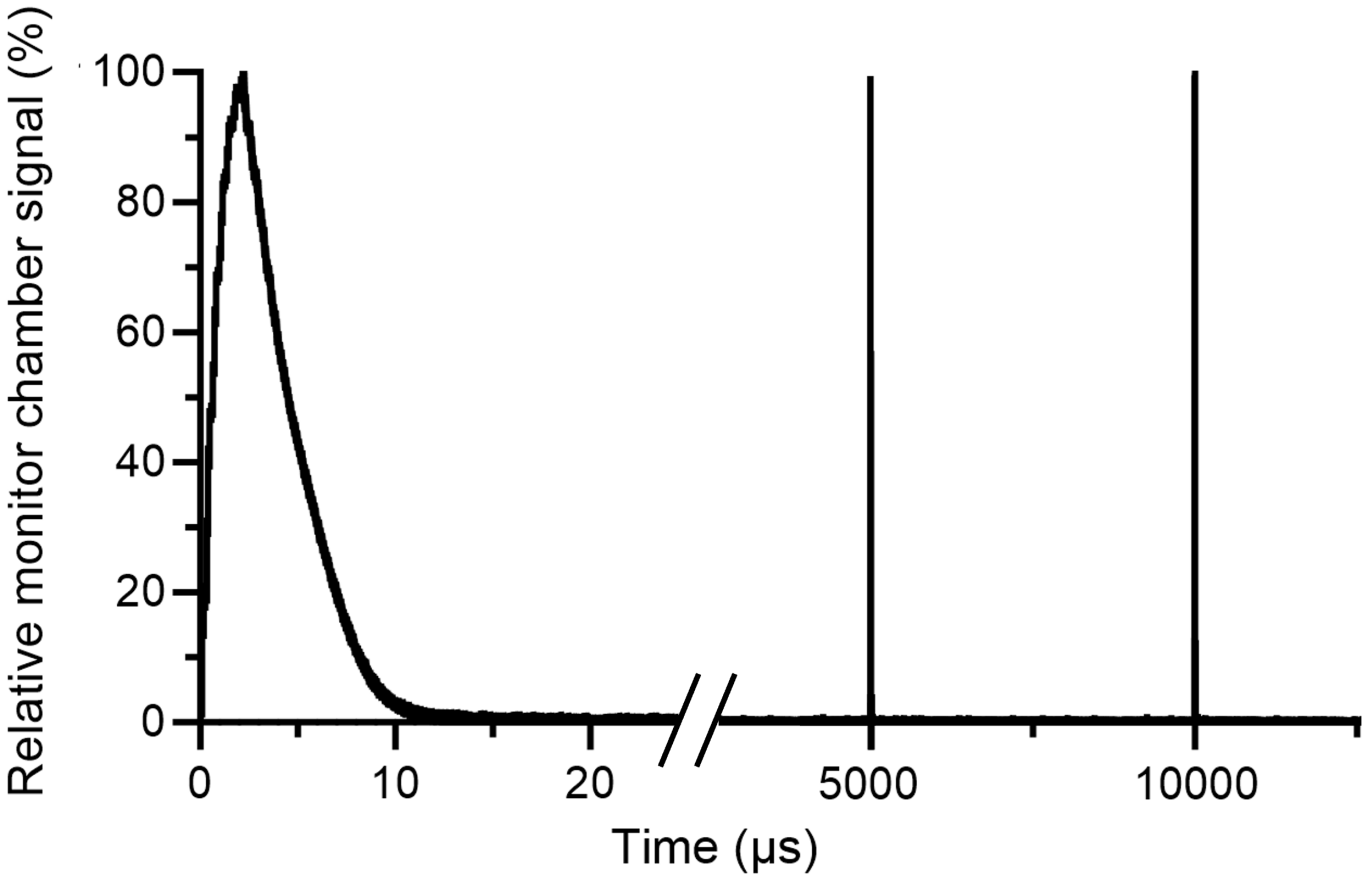
Electron pulses detected by the external monitor chamber. The electron pulses are delivered with a pulse repetition frequency of 200 Hz. After each pulse, the monitor chamber signal is reset within approximately 10 µs, i.e., well before the delivery of any subsequent pulse.

There was a linear relation (r^2^>0.99) between the absorbed dose measured at 2.2 cm depth and the number of MU determined with the external monitor chamber (**Figure 6A**). The deviation between the calculated MU and the absorbed dose (in cGy) was ≤5% for 95% of the total of 40 measurements (**Figure 6B**) and the mean absolute deviation was 3%. There was also a linear relation (r^2^>0.99) between the Farmer-type chamber signal and the number of calculated MU (**Figure 6C**). The associated residuals were ≤5% for all measurements (mean absolute error of 1.1%) (**Figure 6D**). The number of MU determined with the external monitor chamber was also linear (r^2^>0.99) with the mean DPP (**Figure 7**). The number of pulses registered by the pulse counter agreed with the number of pulses detected by the monitor chamber. The temporal structure of the signal from the monitor chamber demonstrated that the signal generated by an electron pulse is reset within approximately 10 µs, i.e., well before any subsequent pulse (**Figure 8**).

**Figure 9:**
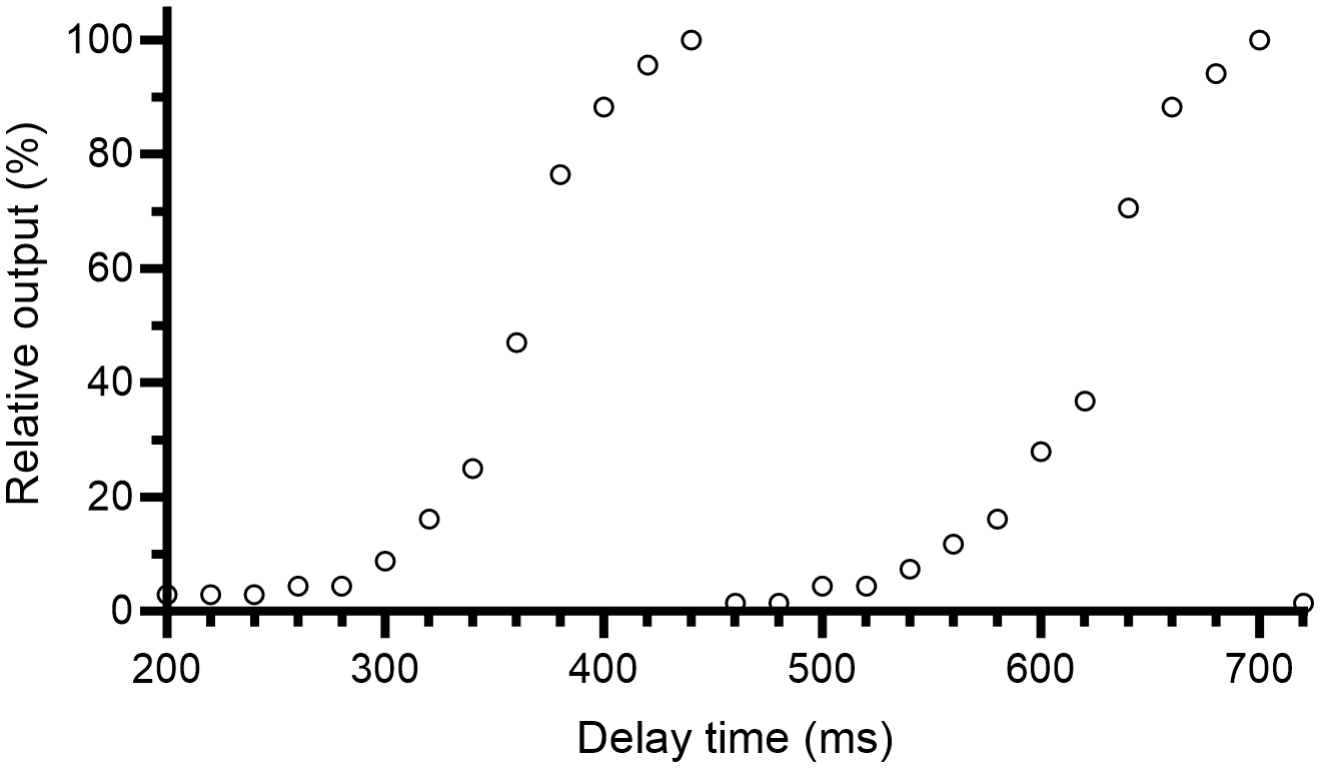
Relative output in the first pulse as a function of the delay time from the detection of a pulse forming network (PFN) signal until the trigger pulses are allowed to pass the optocoupler. The relative output was measured with the Farmer-type ion chamber at 9 cm depth.

The output in the initial pulse was dependent on the PFN delay time (**Figure 9**). The maximal output was found for PFN delay times of 440 ms and 700 ms, where the signal of the Farmer-type ion chamber was 0.068 nC (corresponding to 0.84 Gy at dose maximum at SSD=100 cm). For the total of 50 measurements performed utilizing the PFN synchronization to target the preset number of MU, the absolute percentage error between the delivered number of MU and the set number of MU was 0.8 ± 0.6% (mean ± SD). **Table 1** presents the mean and standard deviation for each of the levels of set MU from 250 to 2500 MU, as well as the delay time used.

**Table 1:** Comparison between delivered and desired number of monitor units (MUs).

**Desired MU**	250	500	750	1000	1250	1500	1750	2000	2250	2500
**Delivered MU** **(Mean ± SD)**	251 ± 5	499 ± 6	750 ± 5	996 ± 3	1251 ± 8	1503 ± 13	1749 ± 10	2003 ± 20	2241 ± 12	2488 ± 21
**PFN delay time (ms)**	580	577	588	620	633	660	680	695	630	630

Five deliveries were performed for each of the preset number of MUs between 250 and 2500 MU (in steps of 250 MU). The PFN delay times were set to target the corresponding desired MU value.

The consistency of the beam control system to interrupt the beam based on the three independent systems (monitor detector, pulse counter, and timer) was confirmed by the safety test (**Table 2**). When all systems were functioning, all deliveries were interrupted based on the number of MU. When the monitor chamber voltage was disabled, all deliveries were interrupted based on the preset number of pulses. When both the monitor chamber voltage was disabled and the pulse counter was removed from the radiation field, all deliveries were interrupted based on the timer.

**Table 2:** Initial safety assessment of the beam control system under different simulated failure modes.

Beam interrupted based on:	Monitor units	Pulses	Time
Successful interruptions	10/10	10/10	10/10
# MU delivered (mean ± SD)	502 ± 7	N/A	N/A
# pulses delivered	N/A	7	N/A
Delivery time	30 ms	30 ms	30 ms

The ability of each of the three systems (monitor detector, pulse counter, and timer) to interrupt the beam was tested independently.

## 4 Discussion

In this study we have introduced and evaluated upgrades to the beam control system for delivering UHDR radiation using our clinical linac. These upgrades have substantially enhanced the safety and precision of the UHDR delivery. Additionally, we have also outlined an improved beam tuning process to achieve a stable output of hundreds of Gy/s at isocenter distance.

The initial version of our beam control system relied solely on a pulse counter to control the beam delivery (8). In this paper, we have augmented safety measures by implementing the ability to interrupt the delivery based on three independent systems: a monitor detector, a pulse counter, and a timer. Whichever of these systems first meets a predetermined criterion will initiate the beam interruption. This ensures that the beam will be interrupted after a preset time calculated based on the preset number of pulses, even if both the monitor detector and the pulse counter should malfunction. In conventional dose rate delivery, if the dual monitor chamber fails to interrupt the beam, the linac’s built-in timer will interrupt the beam, resulting in a minimal overdose to the patient. However, in the case of UHDR irradiation, the overdose to the patient within that timeframe would be deemed unacceptable. Moreover, if the beam is prematurely interrupted due to linac failure during conventional delivery, the treatment can be restarted without any biological consequences, which is not applicable in the context of FLASH-RT. Although we have successfully prevented the linac’s internal interlock system from prematurely interrupting the beam by disabling the built-in monitor chamber signal, the possibility of underdosing cannot be entirely ruled out. In clinical trials, an action plan for addressing premature beam interruptions is crucial, since such events will most likely influence the biological response induced by FLASH-RT. Our improved safety features represent a critical advancement towards the clinical application of FLASH-RT. The safety assessment in this study confirmed the reliability of the beam control system in interrupting the beam based on the three independent systems. Although we here only present the results from an initial safety assessment, which is not enough to prove a clinical reliability, our experience from >1000 exposures have resulted in no observed failure of the pulse counter.

Controlling the beam exclusively on a pulse level does also entail certain limitations concerning the precision of the dose delivery (11, 19). To achieve better control over the delivered dose, our upgraded system utilizes an external monitor detector as a dose monitoring system, continuously measuring the dose delivered by each pulse. Furthermore, we present initial data on the use of PFN synchronization to control the output in the first pulse, enabling us to target the preset number of MUs. This approach allows for a more dosimetrically precise dose delivery. An ideal scenario would involve fine-tuning the output in the final pulse based on the signal collected during the preceding pulses. The linac’s hardware should possess the capacity to adjust the dose in the final pulse using PFN synchronization. However, achieving this would require additional software developments to process the information after each pulse and transmit it to the hardware within the time between pulses. We acknowledge that future work will delve further into this aspect. It should be noted that with a variable DPP, whether variable in the first or last pulse, the reporting of the DPP will be more ambiguous and difficult to standardize.

In the report by Lempart et al., the maximal output achieved was 0.64 Gy/pulse at the position of the cross-hair foil (8). Shortly following that report, an enhanced beam tuning process was implemented, and an updated protocol has been consistently applied in all our radiobiological FLASH studies and veterinary trials. As part of this updated protocol, in addition to adjusting the gun filament current setting to ~7.5 A, fine-tuning of the magneton frequency is performed at the beginning of each day of use in UHDR mode. This refined approach has enables us to achieve outputs exceeding 160 Gy/s or 200 Gy/s at the depth of dose maximum at isocenter distance (SSD=100 cm), with or without the external monitor chamber in the beam path. This highlights the importance of careful beam tuning for optimal output. At conventional dose rates, the Elekta linac’s magnetron consistently maintains a correct operating frequency due to a physical tuner correcting for frequency changes caused by varying temperature. However, when operated at UHDR, there is not sufficient time for the physical tuner to compensate for these temperature-induced changes. As a result, the output becomes highly dependent on the initial position of the physical tuner. The absence of magneton frequency tuning also prevented a stable output in our previous report, leading to a decreased output after approximately 10 minutes (8). This issue has been solved in part also by temporarily reducing the flow of new cooling water to the linac when in UHDR mode. This adjustment ensures consistent and repeatable output, as demonstrated by a SD of 2.2% for film measurements and 0.4% for Farmer-type ion chamber measurements conducted every minute over a 20-minute period. The uncertainty of the film measurements is within the uncertainty of the film (2.7%, 1 SD), indicating a stable output. Additionally, we have achieved a reproducible output, with a SD of 2.6% for film measurements and 2.4% for Farmer-type ion chamber measurements carried out over a 3-months period. The slightly higher SD in the reproducibility in comparison to the short-time repeatability can be explained by the daily variations in the beam tuning. We were also able to verify the stability in the DPP, with an SD of 1.7% as measured by film for 9 individual pulses. It should be noted that this variation was measured with a relative dose in the first pulse close to the mean DPP (~90%). Initial tests have indicated that the variation might increase if the dose in the first pulse is instead low relative to the mean DPP (~5%). This will be investigated further in future studies.

In our current configuration, no scattering foils are present in the beams path and an external transmission chamber is positioned in the beam path, resulting in a Gaussian-shaped beam. If a higher degree of beam flatness is desired, the scattering foils can be reintroduced into the beams path. With only the secondary foil in place, it is still possible to achieve dose rates close to 100 Gy/s at isocenter distance.

The main limitation of this study pertains to the non-ideal behaviour of the external monitor chamber used as a monitor detector. The monitor chamber is subject to severe recombination effects at UHDR, which are corrected for using a logistic model created for the specific chamber, as has been described in previous work (17, 18). This approach to correct for severe recombination effects will induce additional uncertainty in the dose determination. This study demonstrates that the monitor chamber is still useful, nonetheless, a dosimeter with a dose rate independent response is desirable. The beam control system has the capability to employ a variety of detectors that can operate fast enough to interrupt the beam between electron pulses. An alternative method currently under investigation for beam monitoring involves the utilization of beam current transformers (BCTs) (23–25). These devices measure the induced current and provide a real-time temporal display of the total exit charge. Commercially available BCTs have been installed at the Oriatron eRT6 linac (23) and the Mobetron (24, 25). However, further research is necessary to determine the potential of this approach for beam control and interruption. Another limitation of this study is reliance of radiochromic film for absolute dose measurements, as these dosimeters have an intrinsic uncertainty of 3%. Recent advancements on FLASH-compatible ion chambers and calorimeters offer promising alternatives for achieving more accurate absolute dose measurements in the future (26).

## 5 Conclusions

We hereby present an upgraded beam control system and beam tuning process for UHDR electron delivery >160 Gy/s at isocenter distance at a clinical linear accelerator, with improved safety and stability. The system can interrupt the beam based on monitor units and utilize PFN synchronization for improved dosimetric precision in the dose delivery.

## Data availability statement

The raw data supporting the conclusions of this article will be made available by the authors, without undue reservation.

## Author contributions

EK: Conceptualization, Data curation, Investigation, Methodology, Validation, Visualization, Writing – original draft. PW: Investigation, Software, Writing – review & editing. AT: Investigation, Software, Writing – review & editing. BB: Conceptualization, Investigation, Writing – review & editing. SB: Conceptualization, Resources, Writing – review & editing. CC: Conceptualization, Funding acquisition, Investigation, Methodology, Resources, Supervision, Writing – original draft. KP: Conceptualization, Funding acquisition, Investigation, Methodology, Supervision, Writing – original draft.

## Supplementary material

Supplementary Figure 1 EBT3 film calibration curve. Films were calibrated under reference conditions in a clinical 10 MeV beam using the clinical linear accelerator. Films were scanned in a flatbed scanner and the net optical density (netOD) from the red channel was determined. Datapoints represent the average netOD of two film measurements for each of 20 dose levels in the range 0-30 Gy.

Supplementary Figure 2 The film strip used to assess the variation in DPP. The film strip was attached to a circular nozzle on an electric motor operated at 3800 rpm and simultaneously irradiated with 10 pulses using a 1x1 cm^2^ field.
