# Supplementary figures and images for: Beam control system and output fine-tuning for safe and precise delivery of FLASH radiotherapy at a clinical linear accelerator
Source: Front Oncol. 2024 Jan 18;14:1342488. doi: 10.3389/fonc.2024.1342488 (PMC10830783; doi:10.3389/fonc.2024.1342488)

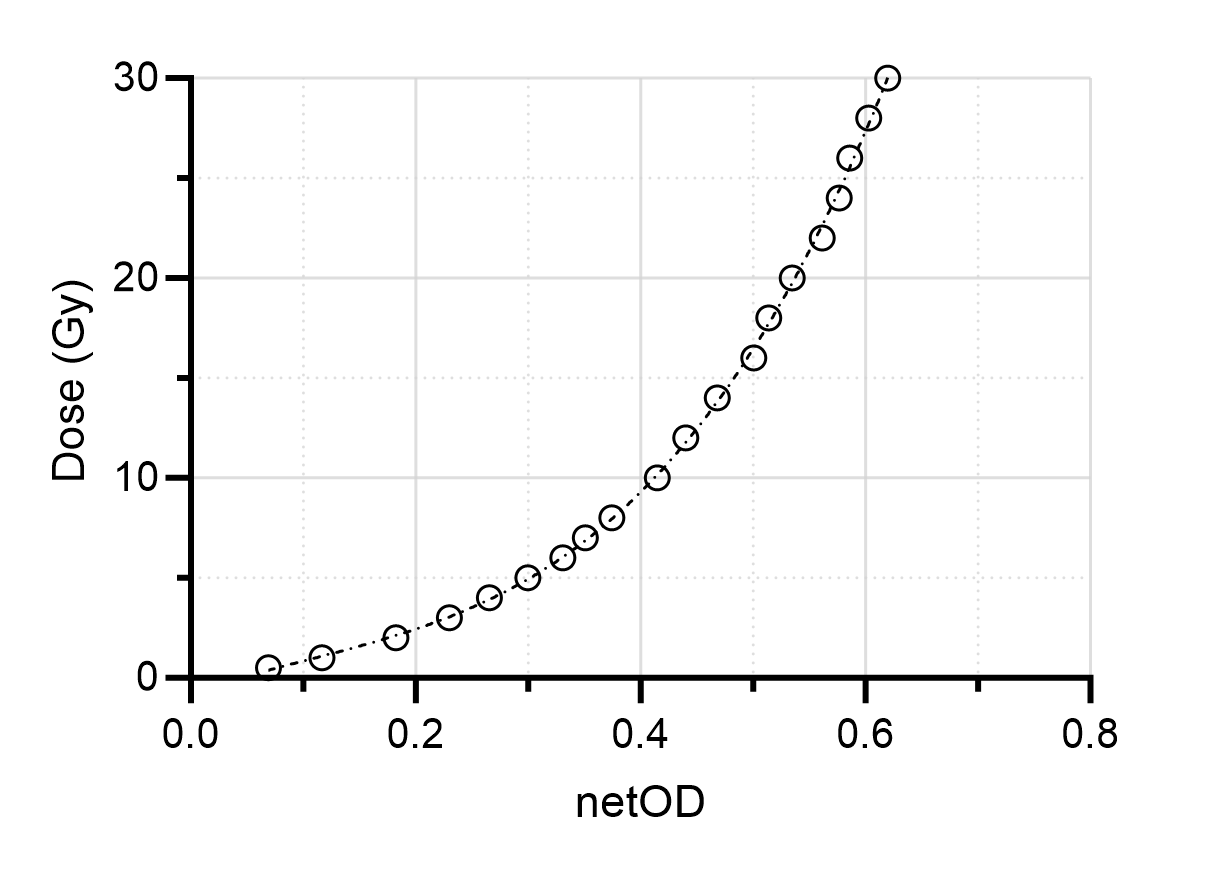

Supplement: Supplementary Figure 1 — EBT3 film calibration curve. Films were calibrated under reference conditions in a clinical 10 MeV beam using the clinical linear accelerator. Films were scanned in a flatbed scanner and the net optical density (netOD) from the red channel was determined. Datapoints represent the average netOD of two film measurements for each of 20 dose levels in the range 0-30 Gy. [file Image_1.tif]

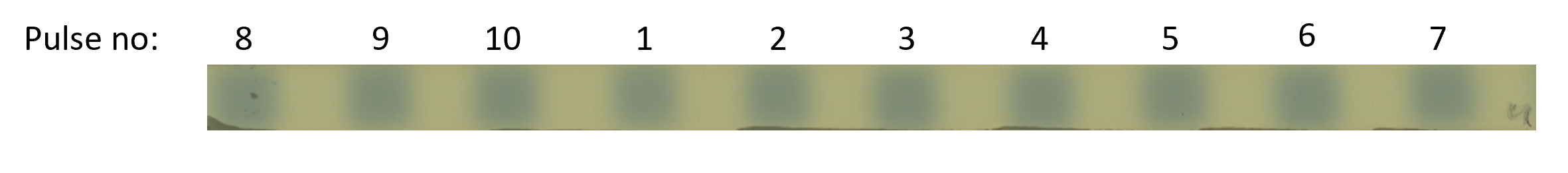

Supplement: Supplementary Figure 2 — The film strip used to assess the variation in DPP. The film strip was attached to a circular nozzle on an electric motor operated at 3800 rpm and simultaneously irradiated with 10 pulses using a 1x1 cm2 field. [file Image_2.tif]
